# American Society for Enhanced Recovery (ASER) and Perioperative Quality Initiative (POQI) joint consensus statement on prevention of postoperative infection within an enhanced recovery pathway for elective colorectal surgery

**DOI:** 10.1186/s13741-017-0059-2

**Published:** 2017-03-03

**Authors:** Stefan D. Holubar, Traci Hedrick, Ruchir Gupta, John Kellum, Mark Hamilton, Tong J. Gan, Monty G. Mythen, Andrew D. Shaw, Timothy E. Miller, for the Perioperative Quality Initiative (POQI) I Workgroup, Timothy E. Miller, Andrew D. Shaw, Michael G. Mythen, Tong J. Gan, Matthew D. McEvoy, Michael J. Scott, Deborah Gordon, Stuart Grant, Julie E. M. Thacker, Christopher L. Wu, Julie E.M. Thacker, Robert H. Thiele, Karthik Raghunathan, CS Brudney, Dileep N. Lobo, Daniel Martin, Anthony Senagore, Stefan D. Holubar, Traci Hedrick, John Kellum, Ruchir Gupta, Mark Hamilton, S. Ramani Moonesinghe, Mike P. W. Grocott, Elliott Bennett-Guerrero, Thomas J. Hopkins, Roberto Bergamaschi, Stuart McCluskey

**Affiliations:** 1 Department of Surgery, Dartmouth-Hitchcock Medical Center, Lebanon, NH USA; 2 Department of Surgery, University of Virginia Health System, Charlottesville, VA USA; 3 Department of Anesthesiology, Stony Brook School of Medicine, Stony Brook, NY USA; 4 Department of Critical Care Medicine, University of Pittsburgh Medical Center, Pittsburgh, PA USA; 5 Department of Intensive Care Medicine and Anaesthesia, St. George’s Hospital and Medical School, London, UK; 6 Department of Anesthesia, UCL/UCLH National Institute of Health Research Biomedical Research Centre, London, UK; 7 Department of Anesthesiology, Vanderbilt University, Nashville, TN USA; 8 Department of Anesthesiology, Duke University Medical Center, Durham, NC USA

**Keywords:** Enhanced recovery, Enhanced recovery pathway, Enhanced recovery protocol, Carepath, Colorectal surgery, Infection prevention, Surgical site infection, Anastomotic leak, Abdominal abscess, Pelvic abscess, Mechanical bowel preparation, Pneumonia, Urinary tract infection, Catheter or line-associated bloodstream infection

## Abstract

**Background:**

Colorectal surgery (CRS) patients are an at-risk population who are particularly vulnerable to postoperative infectious complications. Infectious complications range from minor infections including simple cystitis and superficial wound infections to life-threatening situations such as lobar pneumonia or anastomotic leak with fecal peritonitis. Within an enhanced recovery pathway (ERP), there are multiple approaches that can be used to reduce the risk of postoperative infections.

**Methods:**

With input from a multidisciplinary, international group of experts and through a focused (non-systematic) review of the literature, and use of a modified Delphi method, we achieved consensus surrounding the topic of prevention of postoperative infection in the perioperative period for CRS patients.

**Discussion:**

As a part of the first Perioperative Quality Initiative (POQI-1) workgroup meeting, we sought to develop a consensus statement describing a comprehensive, yet practical, approach for reducing postoperative infections, specifically for CRS within an ERP. Surgical site infection (SSI) is the most common postoperative infection. To reduce SSI, we recommend routine use of a combined isosmotic mechanical bowel preparation with oral antibiotics before elective CRS and that infection prevention strategies (also called bundles) be routinely implemented as part of colorectal ERPs. We recommend against routine use of abdominal drains. We also give consensus guidelines for reducing pneumonia, urinary tract infection, and central line-associated bloodstream infection (CLABSI).

## Summary of consensus statements

### Preventing SSI

1. *Defining incisional vs. abdominopelvic infectious complications*: We recommend reporting and analyzing incisional (superficial and deep) surgical site infections (SSIs) separately from organ space (abscess/leak) SSIs as they are inherently different in both risk factors and consequences.
2. *Drain use and abdominopelvic infectious complications*: We recommend against routine use of abdominal drains. Pelvic drain use should be left to the surgeon’s discretion.
3. *Infection prevention bundles*: We recommend that infection prevention strategies (also called bundles) be routinely implemented as part of colorectal enhanced recovery pathways (ERPs).
4. *Combined mechanical bowel prep (MBP) and oral antibiotics (OAs)*: We recommend routine use of a combined isosmotic MBP with OA before elective colorectal surgery.
5. *Mechanical bowel prep alone*: We do not recommend use of MBP without concurrent OA before elective colorectal surgery.
6. *Isosmotic vs. hyperosmotic mechanical bowel prep*: We recommend against the use of hyperosmotic MBP solutions before elective colorectal surgery.

### Preventing pneumonia and aspiration

7. *Risk assessment*: We suggest preoperative risk assessment for aspiration and pneumonia be routinely implemented in colorectal ERPs.
8. *Optimizing lung function*: We recommend routine use of intraoperative lung-protective strategies during elective colorectal surgery.
9. *Nasogastric tube use*: We recommend against routine use of postoperative nasogastric tube (NGT) drainage after elective colorectal surgery.
10. * Recognizing ileus*: We recommend early recognition and treatment of postoperative ileus. This includes NGT insertion when appropriate.

### Preventing urinary tract infection

11. * Optimal urinary catheter use*: We recommend early urinary catheter removal (within 24 h) after elective colon surgery. Timing of catheter removal after pelvic surgery should be left to the surgeon’s discretion.
12. * Managing early postoperative urinary retention*: We recommend that for patients who fail trial of void, clean intermittent catheterization for 24 h be considered after elective colorectal surgery.

### Preventing CLABSI

13. * Avoid routine central line use*: We recommend against routine central line use during elective colorectal surgery.
14. * Earliest possible removal of central lines*: We recommend that if a central line use is used, it should be removed as soon as possible.

## Background

Colorectal surgery (CRS) patients are an at-risk population who are particularly vulnerable to postoperative infectious complications for a variety of reasons. Primarily, CRS carries a much higher risk of infection than any other clean contaminated case owing to the high bacterial inoculum of the colon and rectum. The colorectal patient population is also characterized by a unique risk factor profile related to underlying diagnoses such as cancer (colorectal adenocarcinoma, anal squamous cell cancer), the presence of active inflammation (diverticular disease and inflammatory bowel disease), and conditions that impair wound healing, such as prior radiochemotherapy, steroids, and malnutrition. Additional complicating factors of CRS include case complexity, perineal wounds, combined multispecialty cases, and prolonged operative times.

**Fig. 1:**
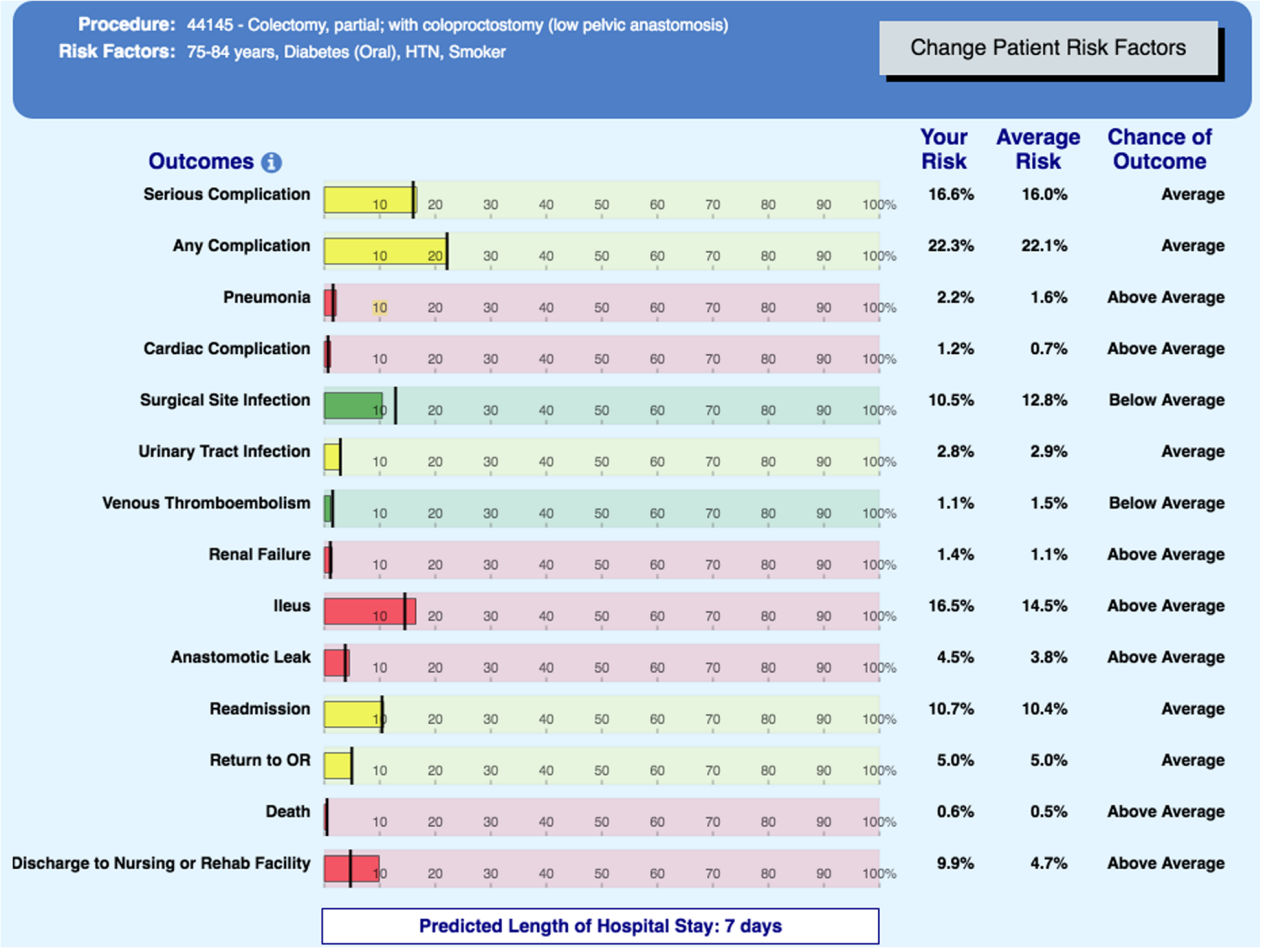
American College of Surgeons Risk Calculator example output

In recent years, the National Quality Improvement Project (NSQIP) (https://www.facs.org/quality-programs/acs-nsqip/program-specifics/participant-use) has allowed for a better understanding of the frequency and risk factors for individual postoperative complications (see http://riskcalculator.facs.org/RiskCalculator/, and Fig. 1, an example of the new calculator output which includes risk of specific infections including urinary tract infection, pneumonia, surgical site infection [SSI], and even anastomotic leak risk). More importantly, NSQIP provides a quality improvement framework whereby individual hospitals and multihospital collaboratives can leverage local, regional, or disease-specific data to improve patient outcomes. A review of recent NSQIP national data reveals an overall complication rate for CRS of 21%, with three fourths of those (15% overall) being infectious complications (Table 1). These hospital-acquired conditions (HACs) range from minor infections including simple cystitis and superficial wound infections to life-threatening situations such as lobar pneumonia or anastomotic leak with fecal peritonitis.

**Table 1:** Definitions of perioperative infections

Type	Rate infections, *n* = 432,756 colorectal procedures^a^ (%)	Median (interquartile range) days from operation to infectious complication	NSQIP definitions^b^	Criteria
Any infectious complication	15.1	–	Composite variable of the below.	N/A
Superficial SSI	5.3	9 (6–14)	Infection involving only skin or subcutaneous tissue of the incision.	Requires symptoms (pain, erythema, swelling, heat) and presence of pus or a positive culture or intentional opening of the wound.
Deep incisional SSI	1.3	10 (6–16)	Infection involving deep soft tissues. Deep soft tissues are typically any tissue beneath the skin and immediate subcutaneous fat, for example, fascial and muscle layers.	Pus must not be from organ space or deep incision spontaneously dehisces or is deliberately opened by a surgeon when the patient has at least one of the following signs or symptoms: fever (>38 °C), localized pain, or tenderness, unless the site is culture-negative direct examination, during reoperation, or by histo-pathologic or radiologic examination radiographic evidence of abscess.
Organ/space SSI	6.4	10 (7–16)	Infection involving any part of the anatomy (e.g., organs or spaces), other than the incision, which was opened or manipulated during an operation.	Pus from a drain that is placed through a stab wound into the organ/space. Organisms isolated from an aseptically obtained culture of fluid or tissue in the organ/space. An abscess or other evidence of infection involving the organ/space that is found on direct examination, during reoperation, or by histo-pathologic or radiologic examination.
Pneumonia	2.5	5 (3–10)	An infection of one or both lungs caused by bacteria, viruses, fungi, or aspiration. Pneumonia can be community acquired or acquired in a healthcare setting.	Requires CXR or CT chest evidence of infiltrate, consolidation, opacity, or cavitation as well as 2 signs, symptoms, or lab values.
UTI	2.6	9 (5–16)	Infection in the urinary tract (kidneys, ureters, bladder, and urethra).	Requires 1 of the following 6 criteria: fever (>38 °C or 100.4 °F), urgency, frequency, dysuria, suprapubic tenderness, costovertebral angle pain or tenderness and a positive urine culture OR 2 of the above criteria and 2 urine cultures or empiric treatment for presumptive UTI.
Sepsis	3.7	7 (3–13)		
Septic shock	2.2	4 (1–9)		
CLABSI	–	–	Not presently included in NSQIP.	

Definitions are available from the 2015 NSQIP Participant User File User Guide: https://www.facs.org/~/media/files/quality%20programs/nsqip/nsqip_puf_user_guide_2015.ashx

^a^Previously unpublished, courtesy of Dr. Holubar, NSQIP 2005–2014, CPT ranges 44xxx–46999

^b^Limited to first 30 days. All definitions may be superseded by surgeon documentation of the infection in the medical record

In the last decade, enhanced recovery pathways (ERPs) have emerged as the optimal approach to perioperative care of colorectal patients. These evidence-based pathways definitively improved postoperative outcomes and are associated with reduced length of stay (LOS) and overall complications (Vlug et al. 2011; Walter et al. 2009). The Perioperative Quality Initiative (POQI) is a collaborative effort that brings together the perspectives of experts representing the perioperative team including anesthesia, surgery, nursing, and other perioperative care providers. In this paper, we will address prevention of infectious complications within an ERP for CRS.

## Methods

On March 4–5, 2016, POQI-1 was held in Durham, NC. Workgroups were composed of healthcare providers (anesthesiologists, surgeons, and nurses) addressing four topics (fluids, outcomes, analgesia, infections). POQI-1 was a consensus-building conference designed around a modified Delphi process in which the group alternately convened for plenary discussion sessions and then retired for small group discussion. The recommendations were developed over 2 days, and consensus was reached around the main issues within each topic. The group chairs and co-chairs were responsible for leading the discussions and delivered a manuscript summarizing the group topic discussions and recommendations and suggestions for future research.

### Preventing surgical site infection

#### Defining incisional vs. abdominopelvic infectious complications

*We recommend reporting and analyzing incisional (superficial and deep SSIs) separately from organ space SSIs (abscess*/*leak), as they are inherently different in both risk factors and consequences*.

In CRS, it is important to differentiate between incisional infections, also called wound infections, which may or may not be associated with a deeper organ space infection that represents an abdominopelvic abscess, anastomotic leak, or enteric fistula. The NSQIP definitions of surgical site infections (SSIs) are shown in Table 1. CRS patients may have multiple incision sites of varying lengths including minimally invasive port sites (<1.2 cm), specimen extraction sites (2–4 cm), new and former ostomy sites (~3 cm), perineal wounds (<6 cm), hand-assisted laparoscopic surgery (HALS) sites (7.5 cm), and traditional laparotomy incisions (8 to 25+ cm). Given their proximity to bowel contents, ostomy wounds and perineal wounds are particularly prone to SSIs.

Superficial SSIs are common at a historic rate of 10 to 25% and are generally recognized clinically as wound erythema, hyperemia, pain, or purulence. Superficial SSIs are generally Clavien-Dindo (Dindo et al. 2004) grade 1 managed by opening the wound and allowing it to heal by secondary intention, often with packing or vacuum dressings. Adjuvant antibiotics are reserved for patients with associated cellulitis >2 cm beyond the wound edge, diabetics, or immunosuppressed patients.

Deep incisional SSIs involve the deep tissues, typically the fascia, in the case of CRS. The development of a deep incisional SSI is particularly morbid. These patients often require operative debridement (Clavien-Dindo grade 3), systemic antibiotics, and are associated with fascial dehiscence and the subsequent development of an incisional hernia. A comprehensive review of all preventative measures for incisional SSIs is beyond the scope of this review. However, the Infectious Disease Society of America Guidelines from 2014 provides an excellent overview (Anderson et al. 2014). Important preventative measures (Table 2) include but are not limited to preoperative smoking cessation, preoperative chlorhexidine showering, clipping for hair removal, prevention of hypothermia, appropriate selection, timing and dosing of prophylactic antibiotics, optimization of glucose control, use of an alcohol-containing skin prep, use of a wound protector, meticulous surgical technique with minimization of GI spillage, adherence to hand hygiene, and reducing unnecessary traffic in the operating room (Yokoe et al. 2014). Infection prevention bundles are discussed further below.

**Table 2:** SSI prevention bundle elements

Phase of care	Element
Preoperative at home	Smoking cessation
Preoperative at home	Diabetes optimization (check and treat HbA1c)
Preoperative at home	Anemia optimization (folate, iron, vitamin C, Venofer)
Preoperative at home	Chlorhexidine showers
Preoperative at hospital	Clipping (not shaving) surgical site
Preoperative at hospital	Chlorhexidine towelettes
Intraoperative	Active warming to prevent hypothermia
Intraoperative	Appropriate (selection, dose, timing) IV antibiotic within 60 min of incision, discontinued within 24 h
Intraoperative	Routine use of a wound protector
Intraoperative	Routine use alcohol-containing skin prep
Intraoperative	Routine intra-op high-concentration supplemental oxygen
Intraoperative	Reduce unnecessary traffic in the operating room
Intraoperative	Routine use of separate fascial closure tray or separate anastomotic tray
Global	Adherence to hand hygiene
Global	Active surveillance program with education, compliance, and feedback
Global	Optimize preoperative glucose control, Maintain blood glucose <180 through POD 2

SHEA/IDSA practice recommendations 2014 (Causey et al. 2011). Note most institutions surgical sub-specialties develop their own bundles to address local issues by selecting a sub-set of the menu of elements listed

Organ space infections, as they pertain to CRS, include abdominopelvic abscesses, anastomotic leak, and enteric fistulae. Relative to incisional infections are less common (<5%) and more morbid (classified as Clavien grades 3 to 5 as fecal peritonitis has a historic mortality rate in the range of 30–50%). Although current mortality rates are much improved, organ space infections are still a very severe complication. Prevention of anastomotic leak centers on constructing a tension-free, airtight, well-perfused anastomosis in a non-contaminated field in a stable patient; low pelvic anastomoses below 10 cm are at increased risk of leak, thus often necessitating fecal diversion via temporary loop ileostomy or colostomy. Historically, the development of an anastomotic leak was felt to be purely technical, related to ischemia, tension, or impaired wound healing. However, clearly, prevention of leaks—especially from an ERP perspective, must take into account potentially reversible patient-related factors such as protein-calorie malnutrition, anemia, and cigarette smoking (Midura et al. 2015).

#### Drain use and abdominopelvic infectious complications

*We do not recommend routine use of abdominal drains. Pelvic drain use should be left to the surgeon*’*s discretion*.

A 2004 Cochrane review found that routine drain use for colorectal anastomoses is of no added benefit (De Jesus EC 2009). More recently, this was confirmed by a subsequent meta-analysis (Zhang et al. 2016). However, it is important to note that these studies did not assign any harm to the drain use either. One concern for the presence of an indwelling drain from an ERP perspective is that it will interfere with the patient’s ability to ambulate independently in the postoperative period; another is potential for infection. As such, the avoidance of drains is one of the primary tenets of ERPs, and we do not recommend using abdominal drains routinely. However, many colorectal surgeons feel that after proctectomy—which results in a large dead space in the most dependent portion of the abdominopelvic cavity—pelvic drains may prevent or help recognize problems such as pelvic hematomas and lymphatic or urinary tract disruption. As such, although pelvic drains should not be used routinely, this decision should be left to the discretion of the surgeon.

#### Infection prevention bundles

*We recommend that surgical site infection prevention bundles be routinely implemented as part of a colorectal ERP*.

Recently, in an effort to maximally reduce postoperative SSI occurrence, SSI prevention bundles have been demonstrated to be effective in reducing SSI rates. Specific bundles have been demonstrated in colorectal, pancreatic resection, and liver resection (Cima et al. 2013; Lavu et al. 2012; Hill et al. 2015). A bundle is a package of various perioperative practices all with the common goal of reducing postoperative infectious complications. Bundles are complementary to, and not mutually exclusive to, ERP. Example practices include preoperative optimization of anemia and diabetes; preoperative chlorhexidine washes; proper antibiotic selection; dosing, and re-dosing; active rewarming; and prompt removal of artificial tubes and lines (Table 2).

The Mayo Clinic Rochester colorectal group (Cima et al. 2013) used a multidisciplinary team to design and implement their bundle. Quality improvement methods including statistical process control charts, as well as descriptive statistics, were used to assess the effectiveness of the implementation of the bundle to reduce SSIs. Bundle implementation was associated with an almost 50% reduction in SSIs (as measured by NSQIP) from 9.8% pre-implementation to just 4% after implementation. The Dartmouth colorectal group implemented a similar bundle and, in a NSQIP series of 119 patients, were able to achieve 1st quartile (exemplary status) with an observed SSI rate of 3.4% vs. a predicted observed rate of 6.1% and expected rate of 9.7% (odds ratio, 0.59, 95% confidence interval 0.34–1.03) (Holubar 2016). Similarly, a study from the Duke colorectal group reported on the sequential effect of first ERP and then their bundle on their SSI rates (Keenan et al. 2015). They found that ERP implementation was associated with a reduced LOS (8.3 vs. 6.6 days, *p* < 0.01), but a reduction in SSI was not observed until after implementation of the bundle, when their SSI rate decreased from 16.1 to 6.3% (*p* < 0.01), and their sepsis rate fell from 11.2 to 1.8% (*p* < 0.01). They also observed the ERP + bundle resulted in a decrease in average cost of admission, from $31,926 to $22,044 (*p* < 0.01). Given the strength of these and a number of other studies, the commonality and costs associated with SSI, and the lack of any detrimental effect of the bundle, we recommend that infection prevention bundles be routinely implemented as part of ERP (Thiele et al. 2015).

#### Combined oral antibiotic and mechanical bowel prep

*We recommend routine use of a combined isosmotic mechanical bowel prep (MBP) with oral antibiotics (OA) before elective colorectal surgery*.

#### Mechanical bowel prep alone

*We do not recommend use of MBP without concurrent oral antibiotics before elective colorectal surgery*.

In this section we will focus on whether or not preoperative bowel preparation, including both MBP and OA, are efficacious in preventing SSI after CRS. We recognize this is a highly controversial topic with divergent practices and opinions between the USA and Europe. At least some of the controversy on this topic results from confusion over the findings of two separate Cochrane group analyses, published within 1 year of each other (reviewed below). In the following sections, we will review the best available literature in an attempt to provide clarity to this topic.

It is imperative to note that all bowel preparations are not the same. MBPs are defined as oral preparations given prior to surgery as a cathartic with the intention of clearing out solid stool; this does not include transanal enemas as, although they are given for the intention of clearing out stool, they are not given orally and are of more limited utility. On the other hand, OAs given often, but not always with MBPs, are intended to decrease the intraluminal bacterial concentration. Historically, as early as 1973, the Nichols-Condon bowel prep was both a MBP and OA regimen (neomycin and erythromycin) and, at the time, reduced the SSI rate from 43 to 9% (Nichols et al. 1973). When interpreting the literature or your own institutional practice patterns, it is imperative to discern whether MBP was used with or without OA.

##### Cochrane #1: intravenous (IV) and oral antibiotics

In 2009, Nelson et al. reported on the efficacy of both IV and oral antibiotics in CRS (Nelson et al. 2009). They found that a combined oral and IV antibiotic prophylaxis was associated with a lower rate of SSIs (RR 0.55, 95% confidence interval [C.I.] 0.41–0.74; RR 0.34, 95% C.I. 0.13–0.87) compared to either IV alone or OA alone, respectively. Their conclusions were that both IV and oral antibiotics should be given routinely and can reduce SSIs by 75%. It is important to note that each of the studies evaluating the use of OA included a MBP. Therefore, it is unknown whether or not OA in the absence of a MBP is efficacious at reducing SSI.

##### Cochrane #2: mechanical bowel prep (MBP) alone

However, in 2011, Guenaga et al. reported on the efficacy of MBPs in >5000 patients (Guenaga et al. 2011). That meta-analysis, which compared MBP to no MBP, and also MBP to rectal enemas, found that there was no increase in complications associated with omission of the MBP. Their conclusion was essentially that MBP could be safely omitted. These findings were echoed by a similar meta-analysis also published in 2011 (Bellows et al. 2011). Other earlier meta-analyses which had similar findings included Pineda et al. and Slim et al. (Pineda et al. 2008; Slim et al. 2009). Nicholson reported a large retrospective cohort study 2011 with congruent findings and recommendations (Nicholson et al. 2011). Note the majority of studies in this review compared *MBP alone in the absence of OA* to the omission of the MBP.

Obviously, the problem with the two different Cochrane meta-analyses is that they are asking different questions. Nelson et al. (Nelson et al. 2010) demonstrates the efficacy of OA (in the presence of a MBP) in addition to IV antibiotics for surgical prophylaxis while Guenaga et al. (Guenaga et al. 2011) demonstrated that a MBP in the absence of OA is not effective in reducing SSI and can be safely omitted. *The finding that MBP alone does not decrease SSI's is not surprising, as a mechanical cleanse in the absence of OA results in bacteria-laden liquid stool that is more likely to contaminate the surgical field*. In addition, the majority of randomized data upon which the practice of routinely omitting bowel preps is based upon did not use combination preps, only MBP without OA.

**Fig. 2:**
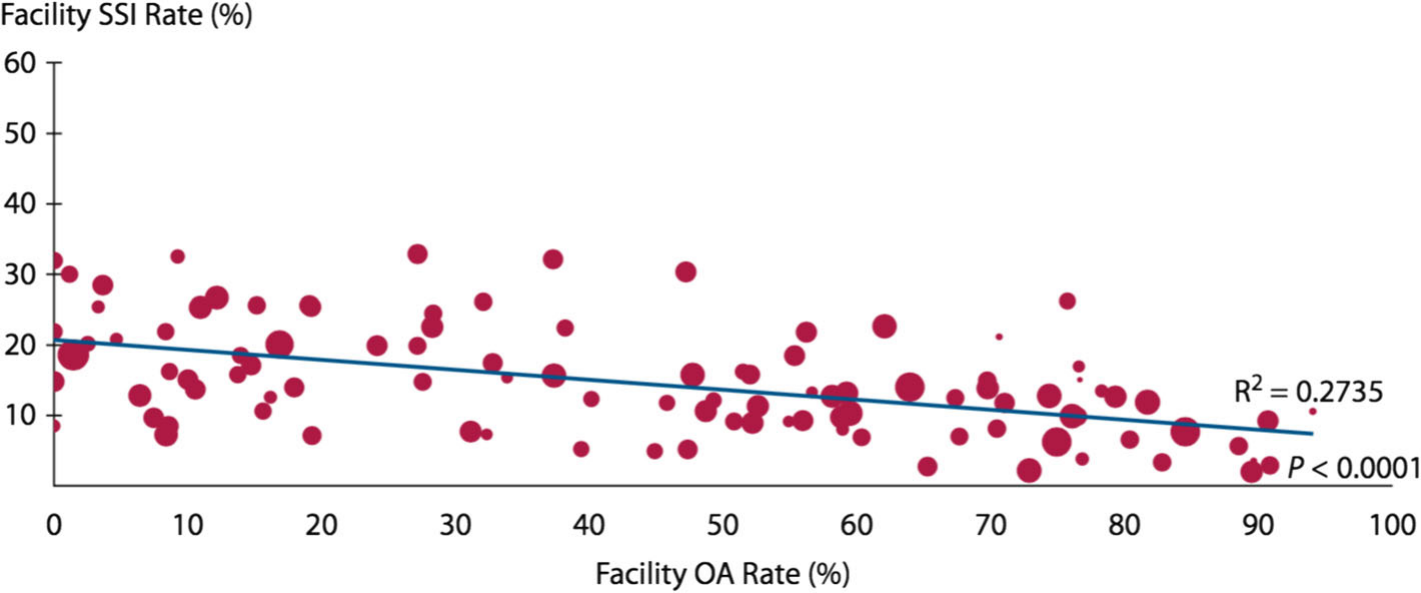
Facility-level surgical site infection rates by oral antibiotic administration. *SSI* surgical site infection, *OA* oral antibiotic. Reproduced with permission from Cannon et al., Dis Colon Rectum 2012; 55: 1160–1166

More recently, in the USA, real-world studies have attempted to definitely answer the question of whether the combination of MBP and OA is more efficacious than MBP without OA using big data. In 2012, Cannon et al. reported a Veterans Affairs SCIP report of 9440 patients (Cannon et al. 2012). They found that MBP (without OA) compared to no MBP (also without OA) had a similarly high rate of SSI (20 vs. 18.1%) but that OA alone resulted in a 67% decrease in SSIs (odds ratio [OR] 0.33, 95% C.I. 0.21–0.5) and OA plus MBP resulted in a 57% reduction in SSI (OR 0.43, 95% C.I. 0.34–0.55); furthermore, they reported a statistically significant strong inverse correlation (*r* ^2^ = 0.27, *p* < 0.0001) between individual hospitals’ OA rate and their SSI rate (Fig. 2). In a follow-up analysis, that group found that OA use was associated with a shorter postoperative LOS and also lower 30-day readmission rates, mostly due to lower rates of infectious complications (Toneva et al. 2013).

**Fig. 3:**
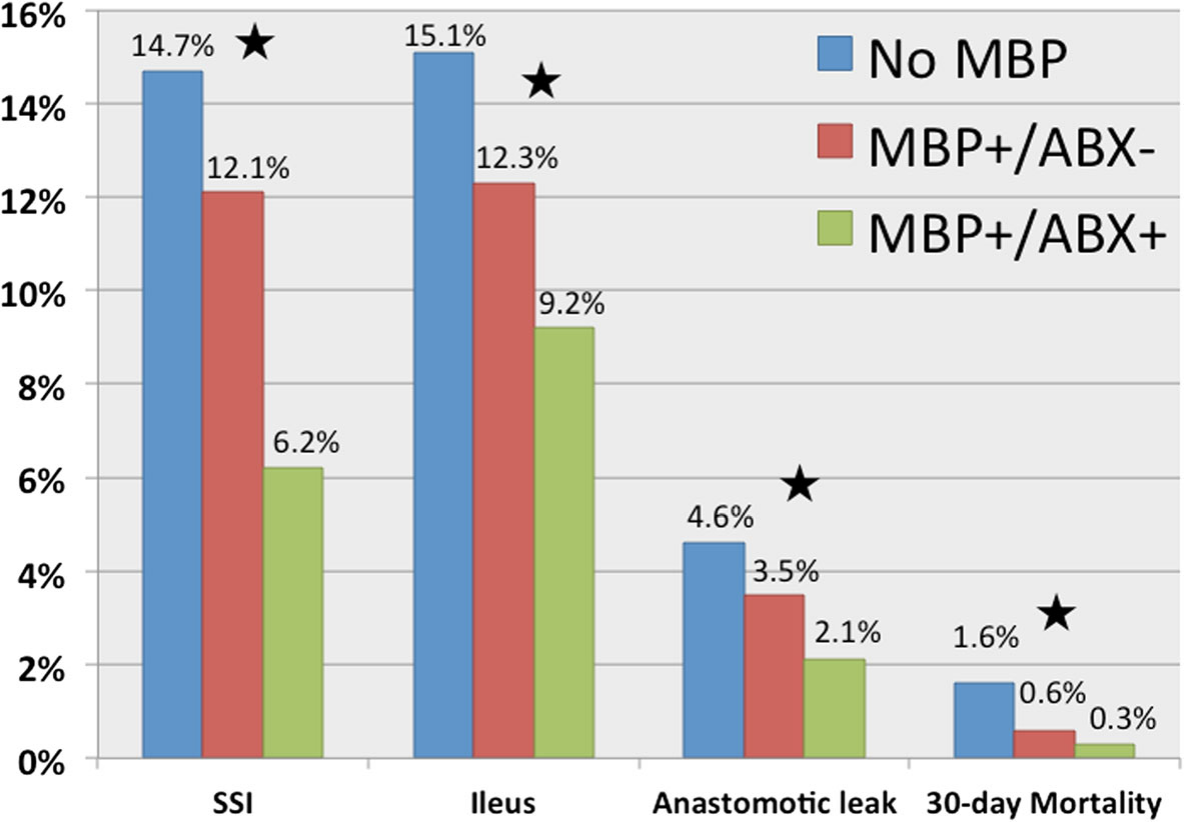
Postoperative complications according to type of bowel preparation. *Black star* = statistical significance, *p* < 0.0001. Adapted with permission from Kiran et al., Ann Surg 2015;262:416 24

Subsequently, in 2015, in another real-world, big data study, Kiran et al. reported a NSQIP study examining >8000 patients and found that the group that had both MBP with OA had a nearly 50% reduction in SSI, anastomotic leak, end even ileus (Kiran et al. 2015). This, although a retrospective study of a prospectively maintained clinical database, likely represents the topical best-single study to date. In this light, *MBP alone and no MBP are equally* ***inferior*** *to combined OA and MBP* (Fig. 3).

In summary, the use of a MBP alone in the absence of OA cannot be recommended. However, the data suggest that a combination of a MBP and OA is associated with the lowest rate of infectious morbidity following CRS. This distinction cannot be overemphasized, as the majority of surgeons in the USA are using a MBP in the absence of OA (Kiran et al. 2015; Moghadamyeghaneh et al. 2015; Morris et al. 2015).

#### Isosmotic vs. hyperosmotic mechanical bowel preps

*We recommend* ***against*** *the use of hyperosmotic bowel prep solutions before elective colorectal surgery*.

It is a common misconception that all MBPs inevitably lead to dehydration and detrimental physiologic effects. This is because many of the phosphate-based solutions initially described in MBP were hyperosmotic solutions. Hyperosmotic preparations (e.g., magnesium citrate, sodium phosphate) exert an osmotic effect, drawing fluid into the bowel. As a result, hyperosmotic solutions are much smaller in volume and typically more palatable to patients. Although better tolerated, these solutions cause significant fluid and electrolyte shifts and can be associated with renal damage (Holte et al. 2004; Ackland et al. 2008; Ackland et al. 2004). For this reason, the use of hyperosmotic bowel preparation solutions within an ERP is not recommended. Table 3 describes the clinical characteristics of various MBP regimens.

**Table 3:** Risks and benefits of various bowel prep solutions

Name	Advantages	Disadvantages
Polyethylene glycol (PEG)	Safe	Large volume, poor taste
Sulfate-free PEG	Safe, better taste	Large volume
Low-volume PEG and bisacodyl	Safe, lower volume (2 vs. 4 L)	Still large volume
Sodium phosphate	Small volume	Electrolyte and fluid shifts, caution in cardiac/liver/renal dysfunction/elderly/dehydrated
Magnesium citrate	Low volume	Electrolyte and fluid shifts

As opposed to the hyperosmotic preparations, the isosmotic solutions of current standard MBP regimens are much better tolerated. Currently, the most widely used cathartic for MBP in the USA is polyethylene glycol (PEG), an isosmotic electrolyte lavage solution that is standardly administered in a 4-L preparation. Because it is a high-molecular weight, non-absorbable polymer, it passes through the GI tract without net absorption or secretion, thereby avoiding significant fluid and electrolyte shifts. The most common side effects associated with PEG solutions are nausea and vomiting, which affect 4–17% of patients on average (Dahabreh et al. 2015). Large-volume PEG preparations include GoLYTELY®, Colyte®, NuLytely®, and TriLyte®. Patients frequently complain about the large volume and salty taste of the solution. As a result, low-volume PEG (32–64 oz, i.e., 1–2 L) preparations combined with other cathartic agents have been developed including MiraLax®, HalfLytely®, MoviPrep®, and BiPeglyte®. While they seem to be effective for colonoscopy, there are few data specifically evaluating the use of these low-volume preparations specific to CRS.

As previously mentioned, isosmotic solutions such as PEG do not share the deleterious physiologic properties as the hyperosmotic solutions. Hendry et al. (Hendry et al. 2008) performed a feasibility study combining the use of oral carbohydrate loading with a MBP in patients undergoing elective left colon and rectal resections, demonstrating that 84% of patients tolerate the preoperative oral fluid/carbohydrate loading in conjunction with the MBP with no untoward effects. Similarly, Thiele et al. (Thiele et al. 2015) demonstrated that a MBP using GoLytely® and OA could effectively be incorporated into a successful ERP. Through implementation of an ERP utilizing a MBP with OA, fluid administration was reduced by 1885 mL during surgery and by 4591 mL over the entire hospitalization resulting in a 2.2-day reduction in LOS and a reduction in overall complications by 48.8% (*p* < 0.0001) including a significant reduction in SSI.

### Prevention of pneumonia after CRS

#### Risk assessment

*We suggest preoperative risk assessment for aspiration and pneumonia be routinely implemented in colorectal ERPs*.

Postoperative pneumonia (PNA) can be broadly classified into two categories: non-aspiration PNA and aspiration PNA. Non-aspiration PNA can be community-acquired PNA (CAP) that occasionally will be recognized immediately prior to surgery and appropriately result in cancelation of general anesthesia. Occasionally, “walking PNA” may go unrecognized and not be diagnosed until postoperatively and is very difficult to differentiate from nosocomial-associated pneumonia (NAP). Nosocomial PNA, a HAC, for postsurgical patients can be further classified as ventilator-associated PNA (VAP) in those who develop it within 48–72 h of endotracheal intubation.

In the setting of ERP, prevention of PNA starts with preoperative risk assessment (Gallart and Canet 2015). The ERP professionals must recognize risk factors for both PNA and for aspiration which are considered pulmonary complications (Yang et al. 2015; Arozullah et al. 2000). Risk factors for PNA include active smoker, former smoker, active pulmonary disease, decreased exercise capacity and/or functional dependency, advanced age, supra-umbilical incision, narcotic use (via respiratory depression), neuroleptic medications, and dementia. Ideally, these risk factors should be identified preoperatively and reversible risk factors addressed as the situation allows.

For example, it is completely ethical for a patient requiring elective sigmoid resection for recurrent uncomplicated diverticular disease to be required to quit smoking for a minimum of 6 weeks preoperatively as smoking is a known risk factor for anastomotic leak (Midura et al. 2015). The recommendation for 6 weeks is based on plastic surgery recommendations and includes a mandatory 6-week period of nicotine abstinence and preoperative testing of plasma or urinary cotinine, a nicotine metabolite (Reinbold et al. 2015). However, for smoking as a risk factor for pulmonary complications, it is generally agreed that any period of cessation can help reduce pulmonary complications, with “the longer, the better” being preferable.

A more extreme example would be a patient awaiting Hartmann’s colostomy closure who also has a giant ventral hernia requiring abdominal wall reconstruction with component separation; in this example, the complication rate is so high and the magnitude of required healing so large that smoking cessation is obligatory. On the other hand, expectation of smoking cessation (or weight loss) may be unreasonable in a colorectal *cancer* patient.

Preoperatively, all CRS ERP patients should receive educational instruction regarding the expectation and motivation for the use of lung function-maintaining adjuncts such as epidural catheters, multimodal non-opioid analgesia, incentive spirometry (IS), coughing, and chest physiotherapy in prevention of PNA and other pulmonary complications. All CRS ERP patients should receive an IS and instructions, ideally preoperatively, or as soon as possible postoperatively (day of surgery upon arrival to PACU or the floor). Some centers provide a pillow with an institutional logo for splinting, while others instruct their patients to bring a small pillow with them from home for this purpose. Finally, when NAP does occur, best available antibiotic regimens should be followed according to infectious disease guidelines and institutional antibiograms.

The other major source of major pulmonary morbidity is respiratory aspiration of gastrointestinal contents, which can be a lethal complication. The relationship between aspiration of gastric contents and aspiration pneumonitis, is well known. The risk factors for aspiration are summarized in Table 4. Awareness of the risk factors for aspiration is the first step in its prevention.

**Table 4:** Risk factors for aspiration (Zargar-Shoshtari et al. 2009)

Patient factors	(a) Full stomach
	· Emergency surgery
	· Inadequate fasting time
	· Gastrointestinal obstruction
	(b) Delayed gastric emptying
	· Systemic diseases, i.e., diabetes mellitus, chronic kidney disease
	· Recent trauma
	· Opioids
	· Raised intracranial pressure
	· Previous gastrointestinal surgery
	· Pregnancy (including active labor)
	(c) Incompetent lower esophageal sphincter
	· Hiatus hernia
	· Recurrent regurgitation
	· Dyspepsia
	· Previous upper gastrointestinal surgery
	· Pregnancy
	(d) Esophageal diseases
	· Previous gastrointestinal surgery
	· Morbid obesity
Surgical factors	Upper gastrointestinal surgery
	· Lithotomy or head down position
	· Laparoscopy
	· Cholecystectomy
Anesthetic factors	Light anesthesia
	· Supraglottic airways
	· Positive pressure ventilation
	· Length of surgery >2 h
	· Difficult airway
*Device factors*	First-generation supraglottic airway devices

Once aspiration has occurred, management should be directed towards supportive modalities and optimizing end organ perfusion. Tracheal suctioning prior to positive pressure ventilation is helpful in preventing aspirated material from damaging the respiratory system. Because aspiration is more likely to affect the right lung secondary to the more vertical angle of the right main bronchus, early chest X-rays will show consolidation in the right side in up to 75% of cases and early bronchoscopy may help prevent distal atelectasis if particulate matter can be aspirated. Aspiration may lead to a variety of clinical conditions, including chemical pneumonitis, bacterial PNA, adult respiratory distress syndrome (ARDS), and complete cardiopulmonary collapse and death. Mechanical ventilation may be required for prolonged periods. The main controversies surrounding treatment decisions involve the decision to use antibiotics and steroids. Antibiotics should only be used if PNA develops, as early antibiotics may lead to the selection of virulent bacteria including *Pseudomonas*. There is no evidence that using steroids either reduces mortality or improves outcome (Muscedere et al. 2008).

#### Optimizing lung function

*We recommend routine use of low tidal volumes, lung recruitment, and other lung-protective strategies during elective colorectal surgery*.

Utilizing protective ventilation strategies with low tidal volumes, optimal amounts of positive end-expiratory pressure (PEEP), and individualized ventilation therapy (which may include spontaneous breathing) have shown improved outcomes for surgical patients.

It has been established in ARDS that lung-protective strategies are best practice, but for elective surgery major abdominal surgery, the role of lung-protective ventilation, although well studied, has had slower adoption and penetration. It is defined as “the delivery of a tidal volume between 6 and 8 ml/kg/predicted body weight, a peak pressure of less than 30 cmH2O, and the use of positive end expiratory pressure of 6-8 cmH2O” (Patel et al. 2016). In a meta-analysis of 15 studies, the authors found a dose-response curve between the size of tidal volume and pulmonary complications (Serpa Neto et al. 2015).

In terms of lung recruitment and PEEP, individualizing PEEP therapy based on respiratory mechanics may reduce the incidence of over-distension and cyclic atelectasis, increase aerated lung available for tidal insufflation, and promote more uniform distribution of mechanical strain (Cressoni et al. 2014). Several mechanics-based PEEP titration strategies have been proposed, including highest respiratory system compliance (Kacmarek et al. 2016), esophageal pressure-guided titration (Talmor et al. 2008), stress index (Grasso et al. 2007), ExPress PEEP (Mercat et al. 2008), and pressure-volume curve lower inflection point (Pflex) (Amato et al. 1998).

There are two concepts for lung recruitment. First, PEEP-maintained lung recruitment refers to the benefit of PEEP to maintain open, at end expiration, the lung parenchyma that has been recruited during the inspiration phase, thus reducing the incidence and severity of atelectasis (Cressoni et al. 2014). The second concept of lung recruitment refers to actual positive pressure that is given to the patient to open up collapsed alveoli. This type of lung recruitment has been advised to be at 40 cmH_2_O for a duration of 40 s (Meade et al. 2008). It occurs in response to hypoxia on current ventilator settings when atelectasis is believed to be the underlying etiology. Thus, there is no set time interval as to how often.

The role of the driving pressure, which is the difference between the plateau pressure and the level of PEEP was studied in a meta-analysis which suggested that driving pressure was associated with the development of postoperative pulmonary complications (odds ratio [OR] for one unit increase of driving pressure 1.16, 95% C.I. 1.13–1.19; *p* < 0.0001), whereas no association was seen for tidal volume (1.05, 0.98–1.13; *p* = 0.179) (Neto et al. 2016).

Finally, spontaneous breathing may be beneficial for patients because this promotes alveolar recruitment, stimulates surfactant production, and attenuates diaphragm disuse atrophy, while avoiding risks of heavy sedation and neuromuscular blockade. However, because spontaneous breathing is not possible in a paralyzed patient undergoing major abdominal surgery, “noisy ventilation,” where different tidal volumes are provided on a breath-to-breath basis (with a coefficient variation of 40%), may be helpful (Spieth et al. 2009). Of note, noisy breathing effort also can lead to high tidal volumes, breath stacking dys-synchrony, regional over-distension, and tidal recruitment, potentiating lung injury risk (Yoshida et al. 2013). Thus, therapy must be individualized based on the patient’s specific needs.

### Nasogastric tubes and early postoperative feeding

#### Nasogastric tube use

*We recommend* ***against*** *routine use of postoperative nasogastric tube (NGT) drainage after elective colorectal surgery*.

Historically, NGTs were used for decompression to theoretically “rest” the anastomosis in the early postoperative period and diminish the risk of anastomotic leakage. The presence of an NGT has clearly been identified as a risk factor for aspiration pneumonia, which is associated with significant morbidity and mortality (Kirby et al. 1995; Langmore et al. 1998; Gomes et al. 2003). In addition, a large Cochrane review encompassing over 5000 patients demonstrated that the routine use of a NGT after abdominal surgery is associated with delayed return of bowel function, increased pulmonary complications, and no difference in anastomotic leak (Nelson et al. 2007). Therefore, the routine use of NGT drainage for decompression after elective CRS is not recommended.

Every attempt should be made to encourage early postoperative oral feeding, which has been demonstrated to be safe and effective following elective CRS (Lewis et al. 2008). Historically, the concern has been that early postoperative feeding would lead to increased rates of aspiration. However, this has not been demonstrated in the literature. In 2003, DiFronzo reported a randomized controlled trial (RCT) on the safety of early feeding in elderly patients who had colon surgery. In this trial of 87 patients over the age of 70, there were no occurrences of PNA (DiFronzo et al. 2003). In 2007, Han-Geurts et al. reported a RCT of 128 patients having elective colorectal or abdominal aorta surgery. Early feeding was not associated with an increased rate of postoperative complications, although the NGT reinsertion rate was 20% in the early feeding group vs. 10% in the nil per os (NPO) group (Han-Geurts et al. 2007). There was no difference in the rate of PNA (7 vs. 10%, *p* = 0.76). Finally, a meta-analysis on this topic of eight studies encompassing 423 patients in the early feeding arm and 426 in the NPO arm found there was no significant difference in the rate of PNA (Andersen et al. 2006).

Alternatively, nasoenteric tubes are occasionally used for enteral feeding in the postoperative period, particularly in patients with significant preoperative malnutrition. There is also evidence that the presence of a nasoenteric feeding tube is associated with colonization and aspiration of pharyngeal secretions and gastric contents leading to a high incidence of Gram-negative pneumonia in patients on enteral nutrition (Gomes et al. 2003). Efforts to feed the patient orally should be exhausted prior to placement of a nasoenteric tube for feeding in the postoperative period. However, a post-pyloric tube is one of the options when a patient does require enteral nutrition (or a percutaneous gastrostomy tube with a post-pyloric extension [“drain me, feed me”] or a percutaneous jejunostomy tube), to reduce the risk of aspiration in the fresh postoperative but critically ill or otherwise unable to take per os (PO) patient. In the colorectal population, bowel dysfunction occurs in approximately 20% and often results in the need for total parenteral nutrition.

#### Recognizing ileus

*We recommend early recognition and treatment of postoperative ileus. This includes NGT insertion when appropriate*.

The prevention, recognition, and treatment of ileus are beyond the scope of this infection prevention article and will be fully addressed in the future in the POQI-2 conference. It is clear that avoidance of prophylactic NGTs and early feeding are both associated with a *lower* mortality (Lewis et al. 2008; DiFronzo et al. 2003; Han-Geurts et al. 2007; Andersen et al. 2006). Although the routine use of NGT decompression is not recommended, this should not be misinterpreted to mean that NGT decompression should not be utilized for the treatment of an ileus. On the contrary, *early recognition of ileus with placement of an NGT is critical to preventing the development of a lethal aspiration pneumonitis*. ERP has been shown to reduce the rates of ileus but does not completely obviate the risk. Any patient with abdominal distention, bilious emesis, and an enlarged gastric bubble on abdominal radiograph requires evaluation by a physician for NGT placement. This is particularly true of elderly patients who are vulnerable to aspiration events. It is critical that the ERP team be vigilant in recognizing and treating ileus and bowel obstruction with nasogastric decompression as needed.

### Preventing urinary tract infection

#### Optimal urinary catheter use

*We recommend early urinary catheter removal (within 24 h) after elective colon surgery. Timing of catheter removal after pelvic surgery should be left to the surgeon’s discretion*.

#### Managing early postoperative urinary retention

*We suggest that for patients who fail trial of void, clean intermittent catheterization for 24 h is associated with a lower rate of urinary tract infection (UTI)*.

**Fig. 4:**
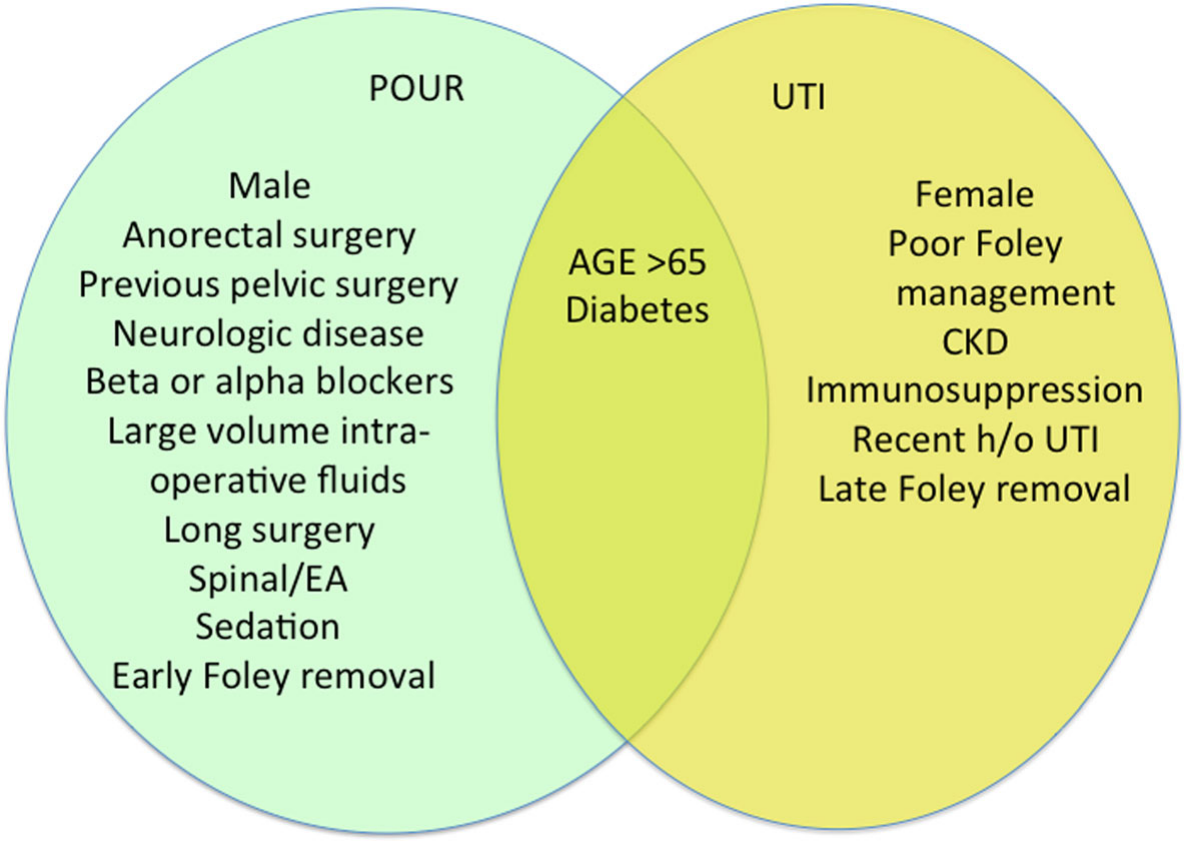
Risk factors for both UTI and POUR

**Fig. 5:**
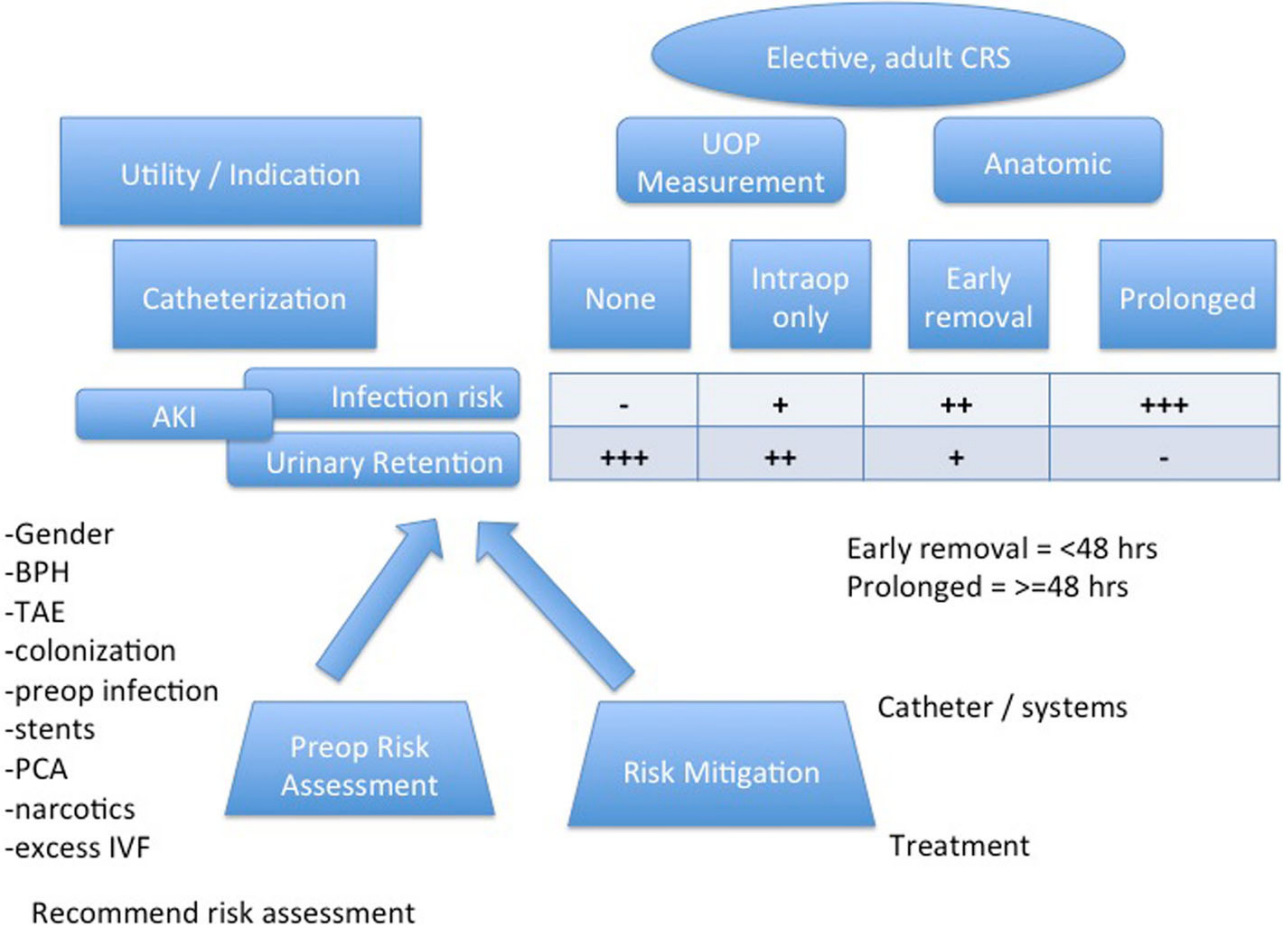
Risks and benefits of urinary catheters

UTIs and postoperative urinary retention (POUR) are both recognized complications of CRS, each occurring in 2–3% of all cases but in substantially higher rates in high-risk patients (Halabi et al. 2014; Kin et al. 2013). Risk factors for both UTI and POUR are shown Fig. 4. Risk for UTI or POUR should guide clinical decision-making for bladder catheterization (Fig. 5). Catheter-associated urinary tract infection (CAUTI) is the most common healthcare-associated infection worldwide (Tambyah 2004). A study in 2565 US hospitals involving 35,904 Medicare inpatients undergoing major surgery in 2001 found that 85% of patients had perioperative indwelling urinary catheters. Of these, 50% had catheters for longer than 2 days postoperatively. These patients were twice as likely to develop UTIs than patients with catheterization of 2 days or less. In multivariate analyses, a postoperative catheterization longer than 2 days was associated with an increased likelihood of in-hospital UTIs (hazard ratio, 1.21; 95% confidence interval [C.I.] 1.04–1.41).(Wald et al. 2008)

However, early removal of indwelling catheters is a recognized contributor to POUR (Baldini et al. 2009). In a prospective observational study in 143 patients, catheters were removed on postoperative day 1 for abdominal surgeries and day 3 for pelvic surgeries. The overall urinary retention rate was 22.4%, and 4.9% developed UTI (Kin et al. 2013). The highest rates of POUR were observed with laparoscopic cases. Spinal or epidural analgesia may increase risk for POUR (Baldini et al. 2009). However, this finding has not been universal. In a US Nationwide Inpatient Sample study of 191,576 laparoscopic colorectal surgeries performed between 2002 and 2010, a 1:4 case-matched analysis was performed, matching for patient demographic characteristics, hospital setting, indications, and procedure type. Epidural analgesia was used in 4102 cases (2.14%) but was not associated with an increased incidence of urinary retention. Conversely, there was a higher rate of UTI (OR = 1.81; *p* = 0.05) (Halabi et al. 2014). Given the retrospective nature of this analysis, it was not possible to control for catheter use and it seems likely there is a trade-off between POUR and UTI based on duration of indwelling catheterization.

One potential contraindication to early catheter removal is in patients known to be high risk for oliguria, which unmonitored, can progress to frank AKI (Drolet et al. 2010; Causey et al. 2011). These specific patients include patients with known CRI, baseline abnormal serum creatinine, creatinine clearance <60, vasculopaths, ICU patients, patients receiving multiple nephrotoxic medications, and those at increased risk for contrast-induced nephropathy (often a transfer patient whose “outside” CT scan is inadequate and repeating would result in an alteration of the surgical plan). Clearly, the average colectomy patient has zero to one of these, and for the majority of colectomy enhanced recovery patients, the catheters should be removed <24 h after placement. This include s removal in the OR for very low risk patients (ex. healthy female, right colectomy).

Thus, we recommend individualization of the decision to use and maintain bladder catheterization based on risk for either POUR or UTI (Fig. 5). As the risks for UTI exceed the risk for POUR, consideration should be given to shorter duration of catheterization. In general, the goal should be to remove catheters by 48 h, as the benefits of continued catheterization are unlikely to exceed the risks after this point. We recommend clean intermittent catheterization to manage POUR after 48 h.

Importantly, various risk mitigation strategies for both CAUTI and POUR have been studied.

Implementation of a “care bundle” including the formation of a multidisciplinary CAUTI reduction task force, formal data collection, staff education for best practices, and new electronic order sets with decision support achieved a reduction in the infection rate per 1000 catheter days from 5.4 to 1.5. Cost savings per 1000 catheter days (±20%) were $4501 ($3600–$5401) (Sutherland et al. 2015). ERPs themselves may reduce UTI (Zargar-Shoshtari et al. 2009; Miller et al. 2014).

A RCT in 239 patients undergoing elective abdominal surgery examined the effect of three doses of trimethoprim-sulfamethoxazole started at urinary catheter removal on UTI. Patients who received antibiotic prophylaxis showed significantly fewer UTIs (5/103, 4.9%) than those without prophylaxis (22/102, 21.6%), *p* < 0.001 (Pfefferkorn et al. 2009). Importantly though, this strategy did not reduce UTI rates to levels seen with early catheter removal but could be an effective strategy for patients requiring prolonged catheterization. Other approaches include novel catheter technology such as silver-impregnated Foley catheters (Leuck et al. 2015).

Strategies have also been examined to reduce POUR. In a retrospective analysis of all men undergoing pelvic surgery between 2004 and 2013 (*n* = 185), patients given 0.4 mg of tamsulosin 3 days prior and after surgery at the discretion of the surgeon had lower rates of urinary retention (6.7 vs. 25%; *p* = 0.03).The authors also found that distal rectal cancer was associated with POUR and tamsulosin may be particularly helpful in that subgroup (Poylin et al. 2015).

### Preventing CLABSI

#### Avoid routine central line use

*We do not recommend routine central line use during elective colorectal surgery*.

#### Earliest possible removal of central lines

*We recommend that if a central line use is used, it should be removed as soon as possible*.

For the purposes of this paper, we define central venous access as placement of a catheter directly into a venous great vessel. The venous great vessels include the superior vena cava, inferior vena cava, internal jugular veins, subclavian veins, and common femoral veins.

Central venous catheters (CVCs) are integral to the care of critically ill patients. CVCs traditionally have been used for three broad purposes: (1) reliable large-bore venous access for fluid and blood product infusion, (2) infusion of medications, and (3) measuring central venous pressures (CVPs). Unfortunately, CVCs are also the leading cause of healthcare-associated bloodstream infections (BSIs) and are frequently implicated in life-threatening illnesses.

Of the approximately 249,000 BSIs that have been shown to occur in US hospitals each year, 80,000 (32.2%) were found in intensive care unit (ICU) settings (Mermel 2000). The economic burden of central line-associated bloodstream infections (CLABSIs) is also quite significant. A single CLABSI episode independently increases length of hospitalization from 7 to 21 days, at an attributable cost of about $32,000 per patient (Stevens et al. 2014). Furthermore, the annual national cost of caring for patients who develop CLABSI is estimated to range from $0.67 to $2.68 billion (Shah et al. 2013).

**Fig. 6:**
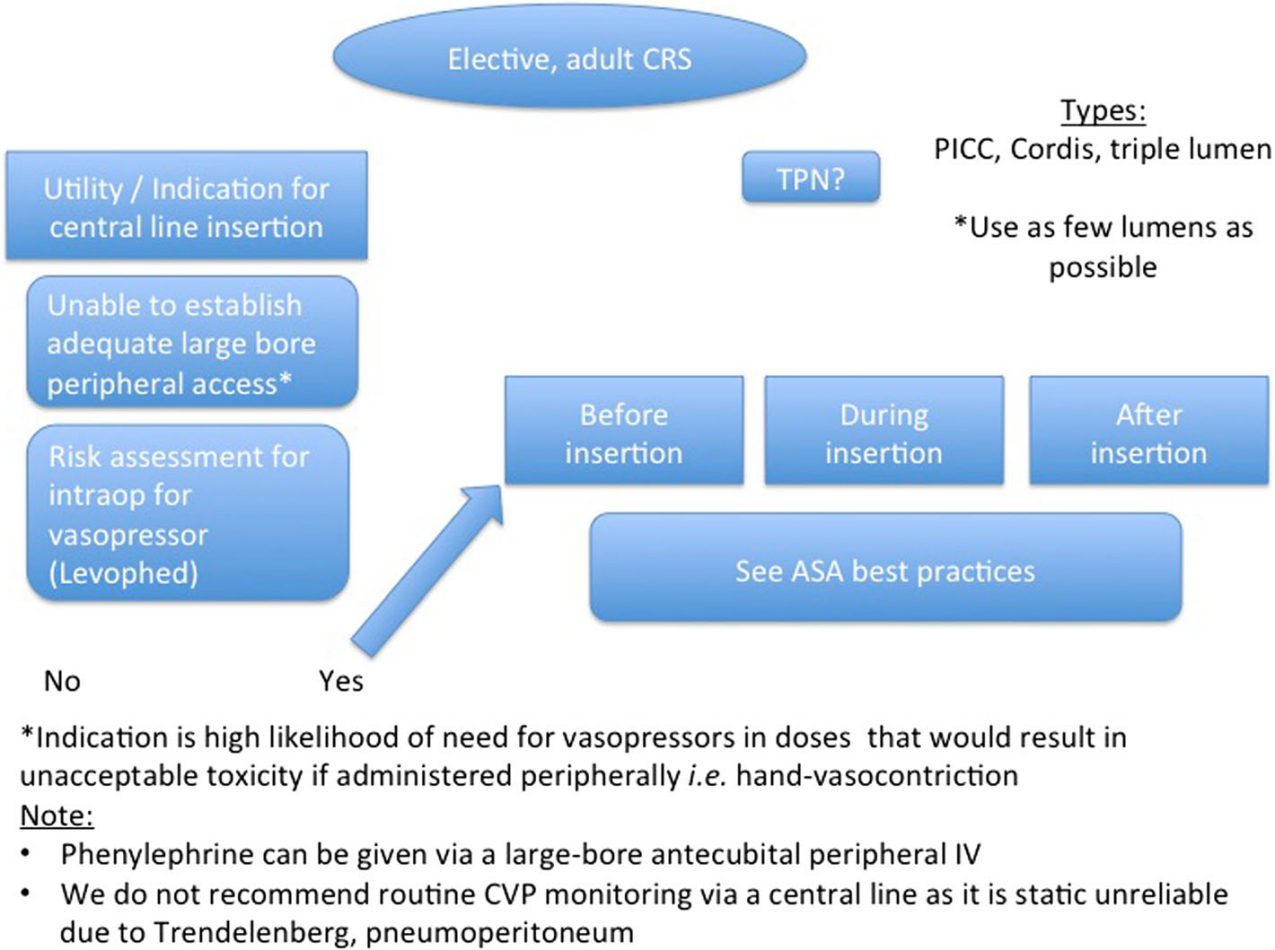
Central line use in colorectal surgery

CLABSIs are potentially preventable through the use of evidence-based practices (Rosendal et al. 2014). We do not recommend the routine use of central venous catheters for CRS, which is not typically associated with significant blood loss or frequent intraoperative complications. Fluids and blood products can be administered peripherally through large-bore (i.e. 16 gauge) IV lines. Furthermore, most drugs can be infused peripherally as well. Those agents needing centrally mediated infusion (i.e., high-dose norepinephrine) would necessitate CVC placement. A risk stratification assessment should occur preoperatively to determine the likelihood of such an infusion intraoperatively, prior to insertion of a CVC (Fig. 6). In terms of hemodynamic monitoring, we do not recommend routine monitoring of the CVP because of the unreliable measurement of CVP in guiding fluid therapy, especially in the presence of increased abdominal pressure and Trendelenburg positioning (Rosendal et al. 2014; Gottlieb and Hunter 2016; Eskesen et al. 2016). Thus, placement of a CVC for monitoring CVP is not recommended. Instead, more modern approaches such as esophageal Doppler, and pulse pressure variation, should be used to guide fluid management, ideally with goal-directed therapy or a zero-balance approach.

Finally, should a CVC be placed, prompt attention should be directed in the immediate postoperative period to determine if there is a continued need for a CVC in the patient. Prompt removal of the CVC and further care via peripheral intravenous lines should be attempted when possible. If patients are discharged with a PICC line, for ongoing IV fluids or TPN, professionals should be aware not only of the risk of infection but of the upper extremity deep vein thrombosis.

### Future directions: prevention of postop SSIs

A number of issues regarding optimal bowel preps remain. Regarding OA with or without MBP, since the bulk of stool weight is bacteria, it is broadly assumed that the efficacy of OA is partly dependent on the mechanical cleansing. To the best of our knowledge, other than the VA-SCIP study mentioned above, few if any studied to date examined the question of OA with or without MBP. The question of *OA without MBP* is ripe for basic-science, translational, or clinical research.

In addition, given the emetogenicity of the standard Nichols-Condon prep (2 g of neomycin and 2 g of metronidazole PO twice), more modern, alternative antibiotic preps are sorely needed. Several interesting studies from Scandinavia used tinidazole, a better tolerated alternative to metronidazole, and doxycycline, to effect low SSI rates (Giercksky et al. 1982; Ofstad et al. 1980). Another interesting, yet unstudied, potential alternative OA bowel prep is rifaximin, a non-absorbable form of rifampin, which has proven efficacy in targeting both GI aerobes and anaerobes (Huang and DuPont 2005; Ojetti et al. 2009). Finally, there is a new FDA-approved product using PEG-infused bars and beverages that allows patients to prep without fasting, which has successfully completed a phase 2 clinical trial for patients undergoing colonoscopy; ultimately, this may prove to be a more tolerable preoperative MBP (personal communication, Dr. Corey Siegel MD).

As stated above, the classic teaching is that leaks are caused mostly by vascular insufficiency, sub-optimal surgical technique, or poor surgical tissues. However, high-quality translational research has emerged suggesting that an infectious etiology secondary to high-collagenase-producing bacteria such as *Pseudomonas* and *Enterococcus* may contribute to anastomotic leak (Shogan et al. 2013; Olivas et al. 2012). This is particularly important as these bacteria may not be covered by a second-generation cephalosporin + metronidazole, which is commonly used for surgical prophylaxis (Alexander et al. 2011). The most recent addition to this growing body of literature suggests that in animals, morphine binding in the bowel is associated with *Enterococcus* colonization, which dovetails with ERPs and multimodal analgesia (Shakhsheer et al. 2016). Reportedly, a large multicenter study using either piperacillin/tazobactam or levofloxacin is being undertaken to address this issue.

Another recent development in preventing anastomotic leaks is the use of indocyanine green (ICG) and near-infrared laparoscopes to assess anastomotic perfusion in real time (Jafari et al. 2015). This results in anastomotic revision in roughly 10% of cases and is a very promising adjunct to reduce anastomotic leak rates. As these units become more widely used, hopefully it will prevent anastomotic leaks and downstream infections.

## Conclusions

In conclusion, although infections are the single most common complication after CRS, ERPs can help mitigate their occurrence and reduce their incidence by avoiding them altogether or hopefully at least reduce their severity and downstream sequelae. The intention of this paper, and the POQI-1 conference, was to develop expert consensus on various target topics. Rather than a prescription, users should use these recommendations as a menu of interventions which should be shared with like-minded progressive, best available evidence-practicing physicians, knowing that individual recommendation of bundle element may or may not be suitable, applicable, acceptable, or affordable at their own institutions. Over the next several years, readers should expect annual POQI papers and POQI-2, which will address ileus among other topics, will be a product of those endeavors.

## Abbreviations

ARDS: Acute respiratory distress syndrome
BSI: Bloodstream infection
CI: Confidence interval
CLABSI: Central line-associated bloodstream infection
CRS: Colorectal surgery
CVC: Central venous catheter
CVP: Central venous pressure
ERP: Enhanced recovery pathway
FDA: US Food and Drug Administration
GI: Gastrointestinal
HAC: Hospital-acquired condition
ICG: Indocyanine green
IS: Incentive spirometry
IV: Intravenous
MBP: Mechanical bowel prep
NAP: Nosocomial-associated pneumonia
NGT: Nasogastric tube
NPO: Nil per os
NSQIP: National Surgery Quality Improvement Program
OA: Oral antibiotic
OR: Odds ratio
PACU: Post-anesthesia care unit
PEG: Polyethylene glycol
PNA: Postoperative pneumonia
PO: Per os
POD: Postoperative day
POQI: Perioperative Quality Initiative
POUR: Postoperative urinary retention
RCT: Randomized controlled trial
SSI: Surgical site infection
US: United States
UTI: Urinary tract infection
VAP: Ventilator-associated pneumonia
